# Level set simulation of focused ion beam sputtering of a multilayer substrate

**DOI:** 10.3762/bjnano.15.61

**Published:** 2024-06-24

**Authors:** Alexander V Rumyantsev, Nikolai I Borgardt, Roman L Volkov, Yuri A Chaplygin

**Affiliations:** 1 National Research University of Electronic Technology - MIET, Bld. 1, Shokin Square, Zelenograd, Moscow, 124498, Russia

**Keywords:** electron microscopy, focused ion beam, level set simulation, multilayer substrate, silicon, silicon dioxide, sputtering

## Abstract

The evolution of a multilayer sample surface during focused ion beam processing was simulated using the level set method and experimentally studied by milling a silicon dioxide layer covering a crystalline silicon substrate. The simulation took into account the redeposition of atoms simultaneously sputtered from both layers of the sample as well as the influence of backscattered ions on the milling process. Monte Carlo simulations were applied to produce tabulated data on the angular distributions of sputtered atoms and backscattered ions.

Two sets of test structures including narrow trenches and rectangular boxes with different aspect ratios were experimentally prepared, and their cross sections were visualized in scanning transmission electron microscopy images. The superimposition of the calculated structure profiles onto the images showed a satisfactory agreement between simulation and experimental results. In the case of boxes that were prepared with an asymmetric cross section, the simulation can accurately predict the depth and shape of the structures, but there is some inaccuracy in reproducing the form of the left sidewall of the structure with a large amount of the redeposited material.

To further validate the developed simulation approach and gain a better understanding of the sputtering process, the distribution of oxygen atoms in the redeposited layer derived from the numerical data was compared with the corresponding elemental map acquired by energy-dispersive X-ray microanalysis.

## Introduction

The focused ion beam (FIB) technique is an effective method for surface nanostructuring. It is based on the local removal of material by sputtering with a narrow beam of, typically, gallium ions. This feature of the FIB method makes it possible to deterministically produce a nanoscale topography on the surface of almost any substrate [1]. FIB milling was originally established in semiconductor technology [2] and materials science applications [3]. Now it is increasingly used for fabrication of complex micro- and nanoscale structures and devices including Fresnel zone plates [4], X-ray lenses [5], optical tweezers [6], and plasmonic antennas [7].

The application of the FIB method is not limited to patterning single-component targets. This technique can also be employed for the modification of multilayer substrates. Depending on the task, the desired properties are achieved by FIB processing of such substrates with different irradiation doses. A low-dose treatment is sufficient for cases that do not require sputtering of large amounts of material. Among such cases are the precise tuning of the magnetic characteristics of thin multilayer films [8], the patterning of 2D materials [9], or the direct introduction of dopants into a solid-state host through recoil implantation [10]. Examples that rely on ion milling include patterning of magnetic multilayers [11], fabrication of optical metamaterials [12], and modification of semiconductor heterostructures [13]. Metal and dielectric layers can be used as hard masks for achieving high resolution and throughput of the FIB nanofabrication process [14]. Modification of integrated circuits [15] is an industrially relevant application of multilayer structure processing.

Effective application of the FIB method requires the creation and optimization of scanning patterns [16–17] as well as the quantitative prediction of ion-induced surface topography [18], which is a challenging task even for single-component substrates. A straightforward analytical description of surface topography is only possible for shallow structures milled under constant sputtering yield conditions [18–20]. For more complex cases, one has to turn to various kinds of computer simulations that considerably facilitate structure fabrication using FIB. For example, surface evolution was studied using segment-based [21], level set [22–24], and Monte Carlo [25] methods. The most commonly studied materials are monocrystalline silicon [21–23] and amorphous silicon dioxide [24–25] because of their technological importance in microelectronics. More complex simulations of multilayer milling, which need to take into account ion beam-induced redistribution and intermixing of atoms from different layers, were considered less frequently. Some of the examples include analytical estimations of the depth of bilayer substrates [26] and cell-based simulations of surface evolution and ion implantation using the Monte Carlo technique [27]. An attempt to simulate the fabrication of structures with simple shape in a multilayer substrate using the level set method was reported in [28].

The aim of this study is to further develop the approach based on the level set method for quantitatively simulating the sputtering of a multilayer substrate under FIB irradiation. The results of the calculations for narrow trenches and rectangular boxes with varying aspect ratios were compared with cross-sectional scanning transmission electron microscopy (STEM) images of experimental test structures fabricated in the silicon dioxide layer covering a crystalline silicon substrate.

## Results and Discussion

### Simulation of sputtering process

#### Application of level set method

**Figure 1:**
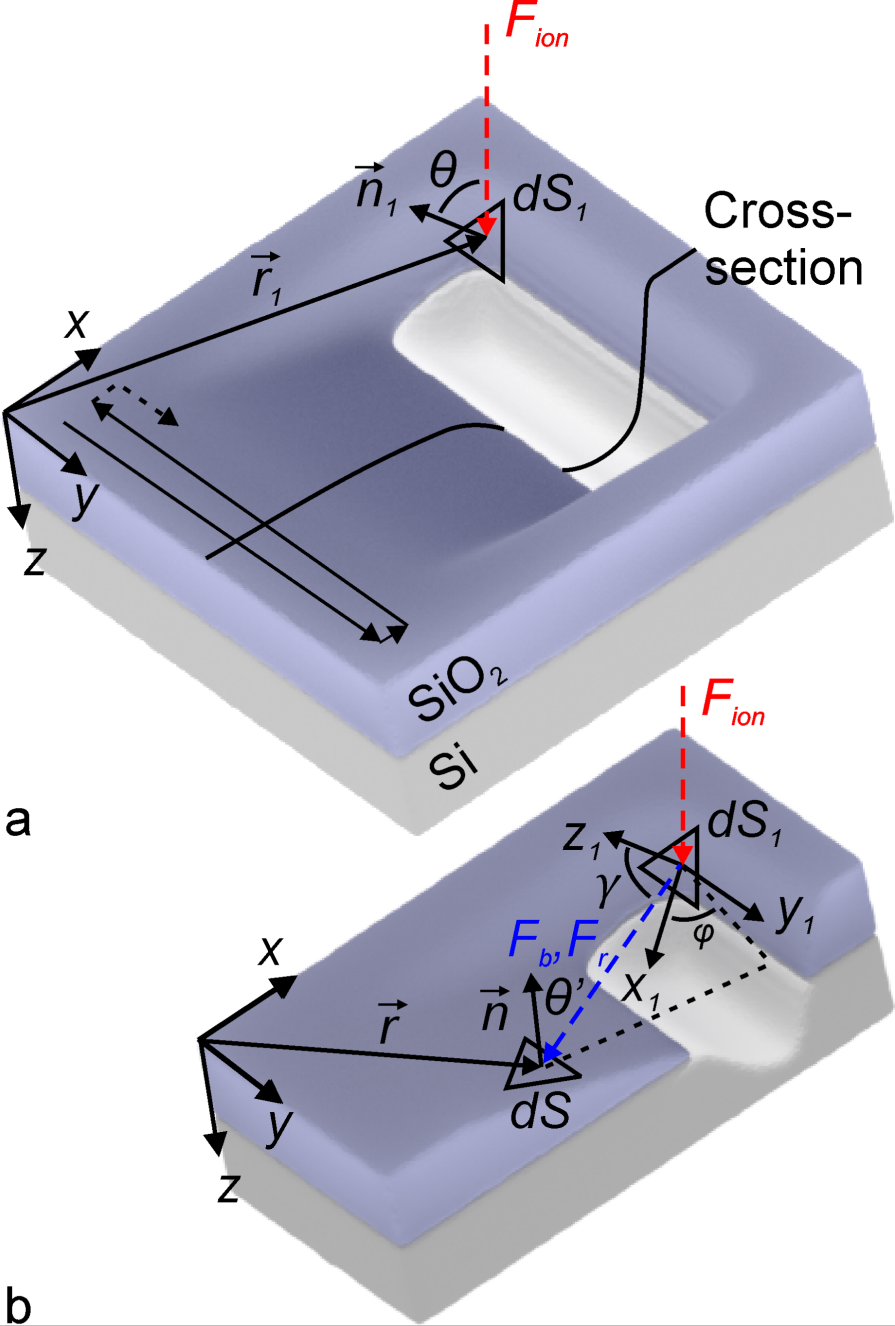
Schematic drawing of the focused ion beam milling induced by the incident ion beam (a) as well as milling and redeposition processes by backscattered ions and sputtered atoms (b). The serpentine scanning path of the ion beam and the position of the cross section are depicted in (a), while the cross-sectional view of the fabricated structure can be seen in (b).

In order to simulate the ion beam milling process by the level set method, the sputtered surface at the moment *t* was described by the expression *z* = *S*(*x*, *y*, *t*), where the *x*-, *y*-, and *z*-coordinates were determined according to Figure 1. The function *S*(*x*, *y*, *t*) determines the distance between the point with coordinates (*x*, *y*) at time *t* from its position at *t* = 0 and is the zero level set of the implicit function Φ(*x*, *y*, *z*, *t*) satisfying the differential equation [29]


[1]

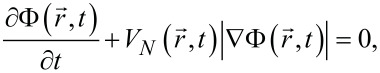



where 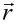 is a vector with coordinates (*x*, *y*, *z*) and 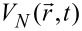 is the displacement rate with which each surface segment moves in the normal direction.

The value of the rate 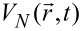 depends on different ion and atom fluxes arising from irradiation by the incident gallium ion beam with the flux 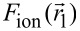. The most important of them is the flux of sputtered particles. For a multilayer structure, it can be presented by the extension of the well-known expression [21] as


[2]

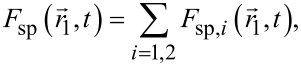



where 
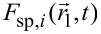
 denotes the flux of sputtered atoms of the *i*-th material with *i* = 1 for SiO_2_ and *i* = 2 for Si. In the case considered, it is equal to


[3]

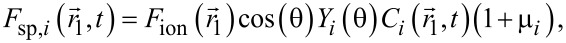



where μ*_i_* = [*Y*_r_*_,i_*(θ)/*Y_i_*(θ) – 1] is the parameter characterizing the difference of the sputtering rates of the *i*-th material in the redeposited and pristine states, the value of which is determined by the corresponding angular dependences of the sputtering yield *Y*_r_*_,i_*(θ) and *Y_i_*(θ). Here, θ is the angle between the direction of incident gallium ions and the local surface normal as shown in Figure 1a, and 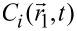 is the atomic fraction of the *i*-th material in the sputtered volume d*V*_1_ in the vicinity of point 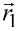. The application of Equation 3 for 
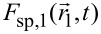
 signifies that, when the silicon dioxide is milled by the ion beam, the relation between the number of sputtered silicon and oxygen atoms corresponds to the stoichiometry of SiO_2_.

In addition to the direct flux of incident ions 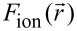, a number of indirect fluxes significantly influences the evolution of the surface [30]. In the following description, we consider the flux of redeposited atoms and fluxes due to backscattering of incident ions.

Strictly speaking, the distribution of atoms of the *i*-th material sputtered from the surface element d*S*_1_ depends on the polar γ and azimuthal φ angles (Figure 1b) as well as on the ion energy *E*. It can be described by the function 
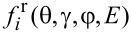
. Since most of the sputtered atoms possess rather low energy with the peak value close to the surface binding energy [31], the sputtering of materials induced by these particles was assumed to be negligible. The energy distribution of sputtered atoms also was ignored in the description of their sticking to the substrate. Therefore, only the angular distribution function 
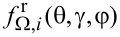
 of the sputtered atoms was of interest, which was defined as


[4] $$f_{\Omega ,i}^{\text{r}}\left(\theta ,\gamma ,\varphi \right)=\int_{0}^{\infty }f_{i}^{\text{r}}\left(\theta ,\gamma ,\varphi ,E\right)\text{d}E.$$

The angles γ and φ of the function 
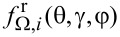
 were determined using an auxiliary Cartesian coordinate system with the origin located at the center of the surface element d*S*_1_ and the *z*_1_ axis directed along the surface normal 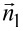 (see Figure 1b). The *x*_1_ axis of the coordinate system was chosen in the beam incidence plane passing through the direction of the ions incidence and the *z*_1_ axis, while the *y*_1_ axis is perpendicular to this plane.

The function 
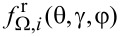
 was normalized as


[5] $$\int_{0}^{\pi /2}\int_{0}^{2\pi }f_{\Omega ,i}^{\text{r}}\left(\theta ,\gamma ,\varphi \right)\sin\gamma \text{ d}\gamma \text{d}\varphi =1,$$

where sin γ dγdφ = dΩ is an infinitesimal solid angle.

Though the large fraction of the sputtered atoms is evacuated from the region affected by the ion beam and does not influence the milling process, some of the atoms can reach the irradiated surface. The flux of such redeposited atoms can be expressed as


[6]

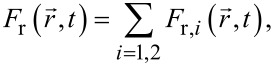



where 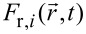 is the flux of sputtered atoms of the *i*-th material equal to


[7]

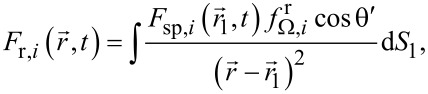



where θ′ is the angle between the normal 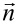 to the surface element d*S* and the vector 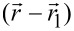 shown by the blue dashed line in Figure 1b, the distribution function 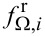 was calculated for the surface element d*S*_1_ (Figure 1), integration was performed over the whole interaction region of the beam with the substrate, and unit sticking probability was assumed for atoms that reached the surface.

In the same way as for the case of sputtered atoms and following [24] we assumed, that the distribution function of the backscattered ions 
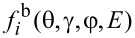
 can be written as product of two independent functions


[8] $$f_{i}^{\text{b}}\left(\theta ,\gamma ,\varphi ,E\right)=f_{\Omega ,i}^{\text{b}}\left(\theta ,\gamma ,\varphi \right)f_{E,i}^{\text{b}}\left(\theta ,E\right),$$

where 
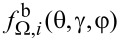
 and 
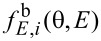
 are angular and energy distribution functions normalized to unity.

Since backscattered ions have a sufficiently wide energy range, their impact on the sample surface causes additional sputtering of the target atoms. Similarly to [24], the function 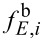 was applied for calculating the angular dependence of the average sputtering yield of the *i*-th material 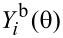 characterizing milling by backscattered ions. The corresponding additional flux of sputtered atoms 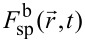 can be described as


[9]

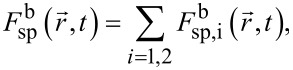



where 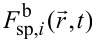 is the flux of sputtered atoms of the *i*-th material equal to


[10]

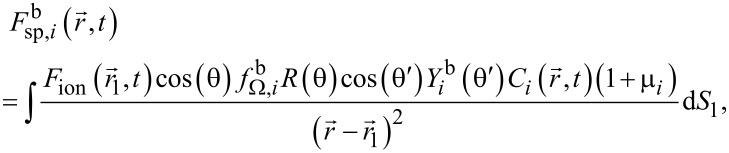



where the function 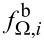 was calculated for the surface element d*S*_1_, and *R*(θ) is the reflection coefficient defined as the ratio of backscattered ions to incident ones.

A fraction of sputtered atoms induced by backscattered ions was redeposited onto the irradiated surface. The flux of such atoms 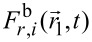 can be calculated using the formula analogous to Equation 7, taking into account that the atoms originate from the surface element d*S* and reach the surface element d*S*_1_ at the angle γ (see Figure 1b).

Using the introduced fluxes and in accordance with [21], the rate 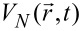 was determined as


[11]

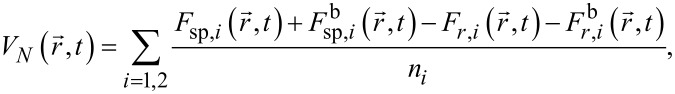



where *n_i_* is the atomic density of the *i*-th material.

The incident ion flux was presented as a sum of two Gaussian functions [32]


[12] $$\begin{array}{c} F_{\text{ion}}\left(x,y\right)\\ =\frac{I}{e}\frac{1}{2\pi \left(\sigma_{1}^{2}+w\sigma_{2}^{2}\right)}\left[\exp\left(-\frac{x^{2}+y^{2}}{2\sigma_{1}^{2}}\right)+w\exp\left(-\frac{x^{2}+y^{2}}{2\sigma_{2}^{2}}\right)\right], \end{array}$$

where *e* is the elementary charge, and *w* is a weighting factor.

The values of the parameters σ_1_ = 53 nm, σ_2_ = 136 nm, and *w* = 0.08 corresponding to our experimental conditions were found using a simple approach presented in [19], which is based on evaluating the shape of milled dot and line structures with different depths.

#### Numerical calculations

Equation 1 was solved numerically on a primary three-dimensional regular rectangular computational grid with a cell (voxel) size equal to 0.3σ_1_ to find the function 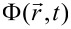. The function *S*(*x*, *y*, *t*) was determined based on 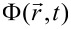 at each time step Δ*t* using the marching cubes algorithm and an irregular mesh of triangular elements [33]. In order to solve Equation 1, the ion fluxes and the displacement rate 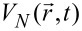 were calculated on the surface *S*(*x*, *y*, *t*) and transferred via nearest-neighbor interpolation to the primary computational grid. The experimental angular dependences of the sputtering yield 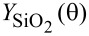 [24] and *Y*_Si_(θ) [34] were used for the calculations of *F*_sp,_*_i_* with μ*_i_* values of 0.15 for SiO_2_ (*i* =1) [24] and 0.3 for Si (*i* = 2) [35].

The function 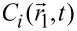 at t=0 was specified by the structure of the Si substrate covered by a SiO_2_ layer irradiated by a focused ion beam. The flux of atoms sputtered from the SiO_2_ layer and the silicon substrate on the milled surface, which form the redeposited layer, caused local composition variations described by the function 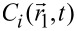, the values of which were stored on the primary rectangular grid introduced for solving Equation 1. The presence of gallium atoms in the redeposited material was taken into account by the coefficient μ*_i_*. The transfer of the values of 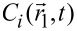 between the rectangular grid and the triangular mesh was carried out as outlined above for the ion fluxes and the displacement rate 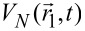.

The variation of the function 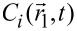 depends on the relation between the fluxes of the sputtered and redeposited atoms. If the condition 
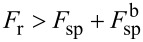
 is satisfied in the vicinity of the point 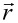 for the sputtered surface element Δ*S*, the total number of redeposited atoms in the cell corresponding to 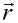 per time step Δ*t* varies by 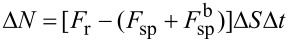, while the number of atoms of the *i*-th material varies by 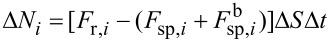.

In order to simplify calculations, the fluxes *F*_sp,_*_i_* and 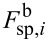 were determined using Equation 3 and Equation 10, without taking into account the parameters μ*_i_* if the fraction of the sputtered material in the cell was below 50%. When this fraction exceeded 50%, the values of the parameters were taken into account, and the cell was marked as containing redeposited material. If in the subsequent calculations the fraction of the redeposited material in the cells dropped below 50%, these cells were marked as containing no redeposited material. The set of the cells with the redeposited oxygen and silicon atoms was employed in the following section where the simulation results were compared with the experimental data. The concentration of the oxygen atoms *C*_O_ in the redeposited material stemming from the sputtering of the SiO_2_ layer was determined as 
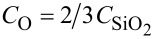
.

For improving accuracy of the calculations and reducing computational efforts, the angular distribution functions 
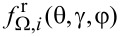
 for incidence angles θ below and higher than 70° were obtained in different ways. For θ ≤ 70° the function 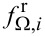 was approximated by the simple expression [21]


[13] $$f_{\Omega ,i}^{\text{r}}\left(\theta ,\gamma ,\varphi \right)=\frac{1}{\pi }\cos\gamma .$$

This dependence increasingly deviates from the results of Monte Carlo simulations as the incidence angle increases [36]. Therefore, for incidence angles exceeding 70° we employed data obtained from Monte Carlo simulations. Similarly, the angular distribution functions 
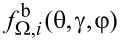
 were found for θ > 70°, while the backscattering process was assumed negligible for incidence angles smaller than 70°.

The values of functions 
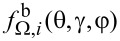
 and 
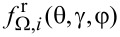
 were tabulated on the basis of SDTrimSP Monte Carlo simulations. The range of incidence angles θ from 70° to 88° was divided into intervals with the step size of 2°, while the hemisphere above the substrate surface was presented as a set of 30 × 120 elementary solid angles with a step of 3° for polar γ and azimuthal φ angles. The average normalized number of atoms (ions) per unit solid angle falling into each elementary solid angle was calculated to determine the functions 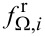 and 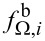. The resulting angular distributions of sputtered atoms and backscattered ions differed slightly for Si and SiO_2_, and an idea of the form of these distributions is given by the plots presented in [36].

Although the functions 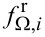 found by Monte Carlo simulations described the angular distribution of sputtered atoms more realistically, the experimental results [37] demonstrated that, at glancing incidence angles, this distribution is shifted by 18° towards larger γ angle values compared to the data calculated using the SDTrimSP software package. In order to account for this discrepancy, when calculating the redeposited atom fluxes *F*_r,_*_i_* for incidence angles exceeding 70°, the auxiliary coordinate system (*x*_1_, *y*_1_, *z*_1_) was rotated in the ion incidence plane around the *y*_1_ axis (Figure 1b). We performed several test simulation runs and found that the optimal rotation angle was equal to 10°, while the rotation by larger angles resulted in underestimation of the depths of the experimentally fabricated structures.

**Figure 2:**
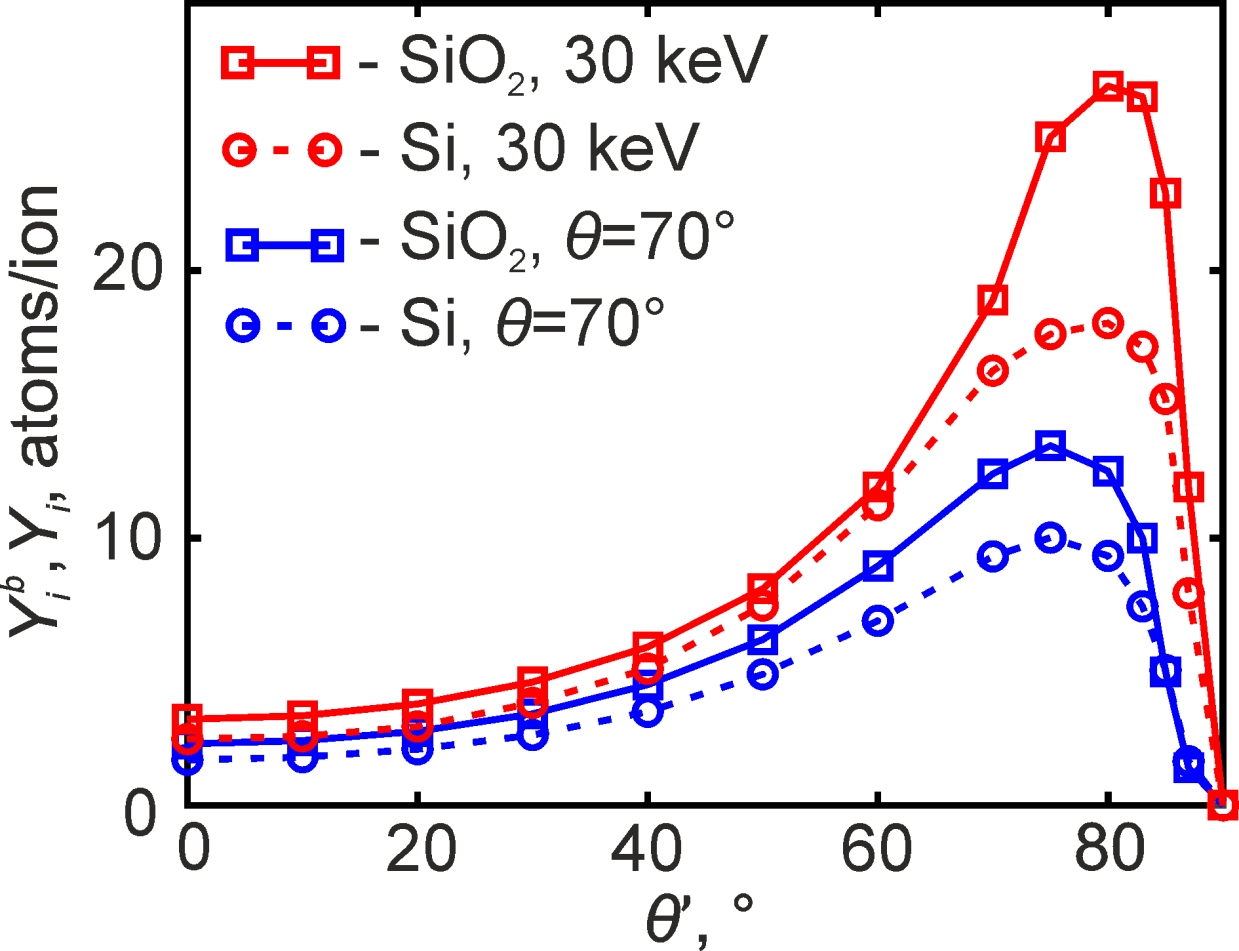
Averaged sputtering yield 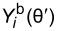 shown together with the dependences of *Y_i_*(θ′) for Ga ions with an energy of 30 keV.

The energy distribution functions 
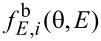
 for incident angles θ > 70° were found by SDTrimSP simulations by dividing the energy range of backscattered ions into intervals with a step of 3 keV and calculating the normalized number of particles falling within each interval. In order to determine the averaged angular dependences of the sputtering yield 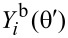 from backscattered ions, their energy distributions described by the functions 
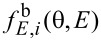
 were taken in account when SDTrimSP Monte Carlo simulations were performed. The obtained dependences 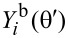 for θ = 70° together with *Y_i_*(θ) for monoenergetic incident ions are shown in Figure 2 for both Si and SiO_2_. Though the angular dependences of the sputtering yield for Si and SiO_2_ are quite different, the corresponding milling rate values, which take into account the atomic densities of the materials, are close to each other.

#### Fabrication and TEM studies of test structures

To verify the simulation results, two sets of test structures of different complexity were fabricated. The first set consisted of three narrow trenches formed in a thin 250 nm SiO_2_ layer by adopting one-dimensional beam scanning along the *y* axis with a pixel spacing of *a* = 38.5 nm. The dwell time value was 0.1 ms, and the number of beam passes was chosen as *M* = 80, 100, and 120, corresponding to ion fluences of 4.4·10^17^, 5.5·10^17^, and 6.6·10^17^ cm^−2^ at the center of the trench, respectively.

**Figure 3:**
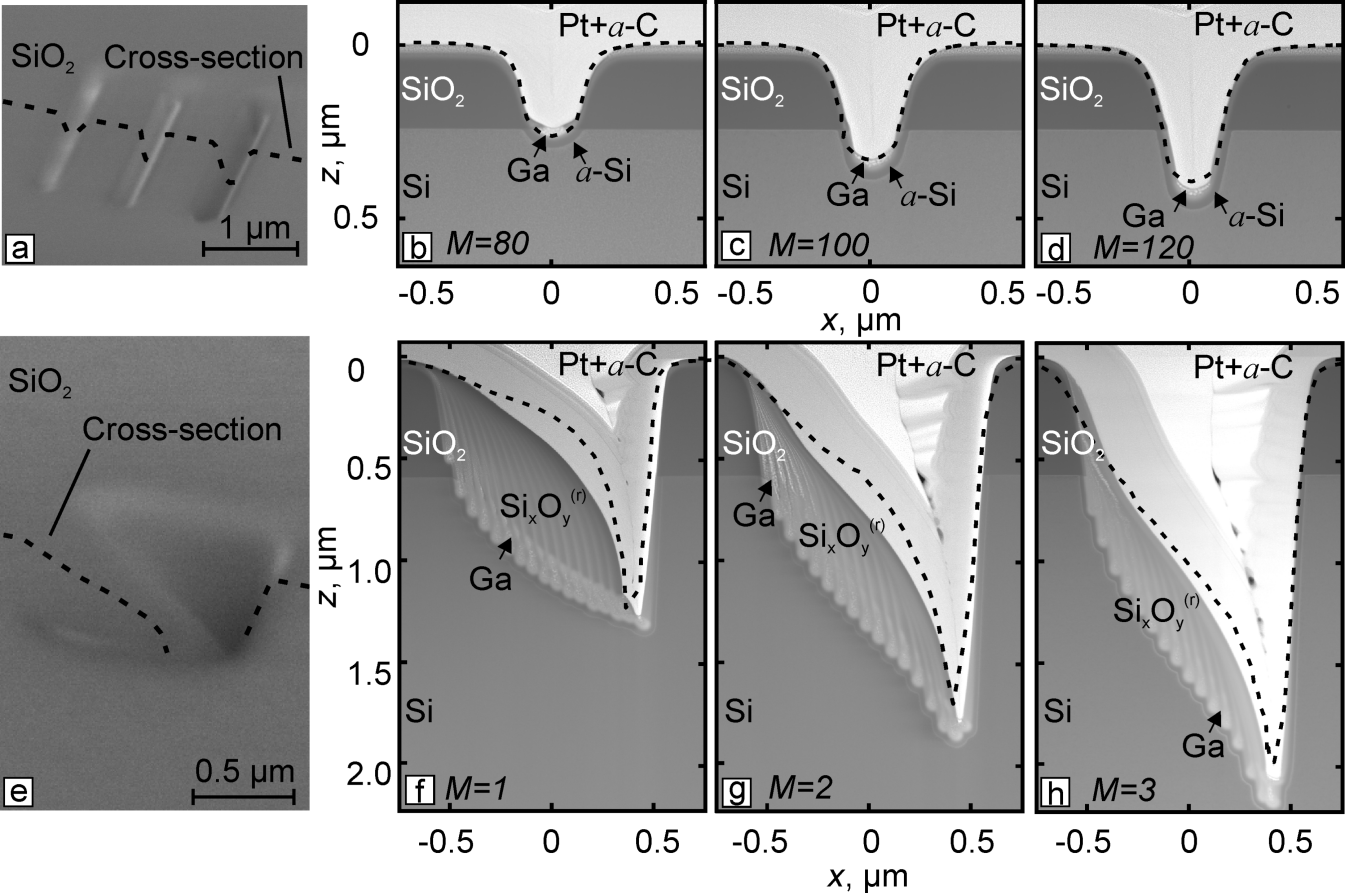
Plan-view SEM (a, e) and cross-sectional STEM (b–d, f–h) images with the superimposed simulation results (dashed lines) of the fabricated narrow trenches (b–d) and rectangular boxes (f–h). The values of *M* indicate the number of ion beam passes.

Figure 3a shows the plan-view scanning electron microscopy (SEM) image of the trenches with a superimposed line depicting the position of the prepared cross section. Figure 3b–d presents the corresponding cross-sectional STEM images of trenches, where the silicon dioxide is visualized as the dark layer. The shape of trenches was mainly determined by the angular dependence of the sputtering yield of the substrate materials and practically uninfluenced by the redeposition process. The aspect ratios of the trenches grew from approximately 0.6 for *M* = 80 to 1.1 for *M* = 120.

The chosen fluence values ensure that the bottom of the trench shown in Figure 3b directly coincides with the interface between the different materials. In contrast, the deeper trenches presented in Figure 3c,d cut through the SiO_2_ layer, and their bottom is located in the crystalline silicon substrate. The small bright regions near the bottom of the trenches correspond to the implanted gallium precipitates, whereas the dark layer surrounding them corresponds to amorphized silicon (*a*-Si).

The second set of structures was fabricated in the serpentine scanning mode with a small number of ion beam passes and pixel spacing values in two orthogonal directions equal to *a* = *b* = 60 nm. The size of the prepared boxes was 1 × 1 μm^2^, and the dwell time value was set at 4.9 ms. The number of the ion beam passes was varied from *M =* 1 to *M* = 3. This corresponded to ion fluences from 7.5·10^17^ to 2.3·10^18^ cm^−2^ and resulted in nanostructures with aspect ratios from approximately 1.0 to 1.7 in a crystalline silicon substrate covered by an approximately 600 nm thick silicon dioxide layer.

The plan-view SEM image corresponding to *M* = 2 and cross-sectional STEM images of the prepared boxes are shown in Figure 3e and Figure 3f–h, respectively. As can be seen from Figure 3e–h, V-like boxes cutting through the SiO_2_ layer with increasing depths are formed at the selected ion fluences. The shape of the boxes is more complicated and significantly influenced by redeposition of the sputtered material, especially in the case of a single ion beam pass (Figure 3f). During the formation of the boxes, sputtered material from both SiO_2_ layer and Si substrate was redeposited in a noticeable amount on the left sidewall of the structure, resulting in its characteristic convex surface shape. Clearly visible, almost vertical stripes in Figure 3f–h are primarily due to variations in the concentration of heavier gallium atoms caused by the ion beam movement during the fabrication of the boxes (see Figure 1a). Each stripe in the image corresponds to one of the line segments oriented along the *y* axis in the serpentine scanning pattern.

The composition of the redeposited 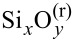 layer varies along the *z* axis direction, and nanoscale precipitates of gallium atoms, visible as small bright spots in Figure 3f–h, appeared in the redeposited layer. Comparing the left sides of the box in Figure 3f–h, it can be seen that the relative amount of deposited material decreased after the second and third ion beam passes because of the higher sputtering rate of the redeposited layer compared to the pristine materials. Figure 3f–h also shows the thin vertical redeposited layer on the right sidewall of the boxes, which influences their surface shape and depth.

**Figure 4:**
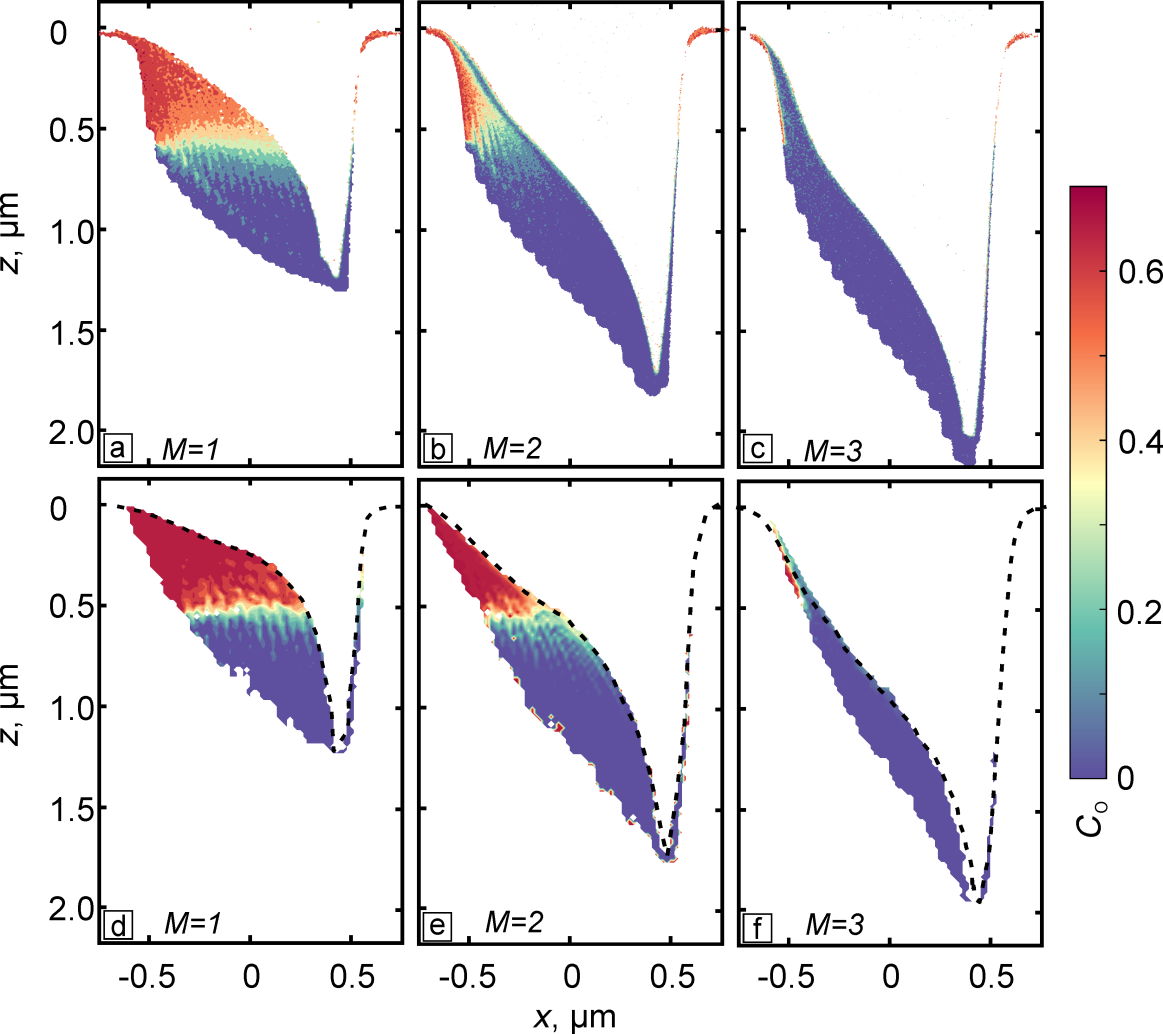
Experimentally obtained (a–c) and simulated (d–f) elemental maps of the oxygen atom distribution in the redeposited material for the structures fabricated using different numbers of ion beam passes, *M*. The color bar indicates the oxygen concentration in the redeposited material.

Cross-sectional specimens of the boxes were also examined using energy-dispersive X-ray microanalysis with a focus on the distribution of oxygen atoms in the redeposited layer, which was recognized in the elemental map acquired for silicon, oxygen and gallium by the presence of Ga atoms. Subsequent analysis of silicon and oxygen concentrations was performed only in the gallium-enriched region, and its results are shown in Figure 4a–c using maps for O. The presented color scale allows for estimating the concentration of oxygen atoms *C*_O_ in the redeposited material, calculated without taking gallium atoms into account. The red color corresponds to the relation between silicon and oxygen atoms as in chemically pure silicon dioxide, while the blue color marks a region in the redeposited layer where oxygen atoms are absent.

Figure 4a–c confirms the inhomogeneous distribution of silicon and oxygen atoms in the redeposited material. The fraction of oxygen atoms reached maximum values closer to the top surface of the processed sample, while the oxygen concentration gradually decreased in the direction parallel to the *z* axis. Inclined stripes in the direction almost parallel to the *z* axis are clearly visible in the volume of the redeposited material. Here, they indicate a nonuniform distribution of oxygen and silicon atoms, rather than gallium ions as seen in STEM images in Figure 3f–h.

The appearance of these stripes was caused by successive milling of the SiO_2_ layer and Si substrate on the right side of the boxes, when the ion beam was shifted by one step along the *x* axis. As a result, the fraction of sputtered oxygen atoms arriving on the left side of the boxes was periodically changed in the upper part of the redeposited layer, as can be clearly seen from Figure 4a. The abrupt boundary between the SiO_2_ layer and the Si substrate was responsible for a sharp change in the composition of the redeposited material along the *z* axis. Figure 4b,c also demonstrates that the composition of the redeposited layers became more and more homogeneous, as the number of passes increased.

#### Comparison between experiments and simulation

Calculated profiles of the simulated structures were superimposed onto the experimental STEM images. Figure 3b-d indicates that the simulation can predict the depth and shape of experimentally fabricated trenches. As is seen from Figure 3f–h for the case of rectangular boxes, the calculated profiles overestimated the amount of the redeposited material on the left sidewall of the structures, while their depth and right sidewall shape were reproduced with reasonable accuracy by the simulation.

Figure 4d–f presents simulated maps of the oxygen atom distribution. Since the voxel size in the simulation was larger than the size of a single pixel in the experimental composition maps, the fine details that were visualized in the experimental maps were not resolved in the calculated ones.

The comparison between experimentally acquired and numerically calculated maps shown in Figure 4a–c and Figure 4d–f, respectively, demonstrates their qualitative similarity. The simulated maps reproduce the amount of the redeposited material, the distribution of oxygen atoms along the *z* axis, as well as the pattern of stripes discussed above with reasonable accuracy. The main difference between Figure 4a and Figure 4d are the shape of the left sidewall of the redeposited layer and some discrepancies in the amount of the redeposited material near the bottom of the boxes. To a lesser extent, similar differences are revealed when comparing Figure 4b and Figure 4e, or Figure 4c and Figure 4f.

A not quite adequate description of the redeposited layer shape in the calculated maps may be attributed to the fact that secondary milling caused by the sputtered atoms was ignored during simulations. Most of these atoms possessed low energy, as it was mentioned earlier. However, as discussed in [38], they occurred in large numbers because of the high sputtering yield, when incident ions irradiated the nearly vertical right sidewall of the prepared boxes with an incidence angle θ close to where the function *Y_i_*(θ) reached its maximum (see Figure 2). This issue will be considered in further studies.

## Experimental

### Methods

Test structures were experimentally prepared using a dual beam workstation Helios Nanolab 650. Both types of test structures were fabricated using 30 kV accelerating voltage and an ion beam current of *I* = 900 pA. The chamber pressure was not higher then 10^−6^ mbar. A layer of platinum and amorphous carbon (Pt + *a*-C) was deposited onto the manufactured structures to protect the surface. Cross section specimens for transmission electron microscopy investigation were prepared using in situ lift-out [39]. Final polishing was performed at the glancing incidence angles of the ion beam through the gradual decrease of the accelerating voltage from 30 to 2 kV.

The STEM micrographs were obtained by means of the high-angle annular dark-field detector of a Titan Themis 200 transmission electron microscope at 200 kV accelerating voltage. Chemical analysis of the selected specimen areas was carried out by energy-dispersive X-ray spectroscopy with the use of a four-quadrant silicon drift detector installed within the microscope.

### BCA Monte Carlo simulation

Angular and energy distributions of sputtered atoms and backscattered ions were studied numerically employing the SDTrimSP software package [40]. The calculations were carried out in the static mode using the krypton–carbon interaction potential [41] and the default surface binding model. For silicon atoms, both in chemically pure silicon and in silicon dioxide, the surface binding energy was set equal to 4.72 eV, while an oxygen surface binding energy of 2.58 eV was chosen according to [40]. The number of calculated ion trajectories for each angle θ was equal to 10^7^. The electronic energy loss was described by the equipartition of Oen–Robinson [42] and Lindhard–Scharff [43] models. The atomic density of amorphous SiO_2_ was assumed as 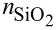 = 6.9·10^22^ cm^−3^ in accordance with [44].

## Conclusion

In this study, an approach based on the level set method was extended for simulating the milling of a multilayer substrate by a focused ion beam, taking into account the backscattering of incident ions and the secondary sputtering of the redeposited material. The developed approach was applied to simulate narrow trenches and rectangular boxes with different aspect ratios, which were milled into a silicon substrate covered with a silicon dioxide layer. Monte Carlo simulations were performed to obtain tabulated data for the angular distributions of the sputtered atoms and the backscattered ions for ion incidence angles exceeding 70°, instead of the usually used cosine distribution, as well as for calculating the average angular dependences of the SiO_2_ and Si sputtering yields induced by the backscattered ions.

The simulation results were compared with experimental data for the FIB-fabricated structures. The calculated profiles were superimposed onto the cross-sectional scanning transmission electron microscopy images of trenches and boxes. It was demonstrated that the simulation enabled a prediction of the shape of narrow trenches with reasonable accuracy. The calculated profiles for the rectangular boxes slightly overestimated the amount of the redeposited material. Further comparison of oxygen atom distributions in the redeposited material extracted from the experimental and simulated chemical maps revealed some discrepancies in the shape of the redeposited layer obtained from simulation and experiments. These discrepancies can presumably be eliminated if material milling by the sputtered atoms is taken into account in the simulation.

Finally, the findings of the work demonstrate that the proposed simulation approach can accurately predict the shape of structures fabricated by a focused ion beam in multilayer substrates and can be useful for different practical applications.

## Data Availability

The data that supports the findings of this study is available from the corresponding author upon reasonable request.
